# From cultural realization to psychological resilience: traditional Chinese folk songs, well-being, and intergenerational knowledge transmission in indigenous ethnic minority communities

**DOI:** 10.3389/fpsyg.2026.1977815

**Published:** 2026-10-21

**Authors:** Haichuan Tang

**Affiliations:** Shandong University of Arts, Jinan, Shandong, China

**Keywords:** cultural realization, indigenous ethnic minority communities, intergenerational transmission, moderated mediation, psychological resilience, traditional folk songs, well-being

## Abstract

**Introduction:**

Folk songs such as Zhuang antiphonal singing, Dong Grand Song, Miao Feige, and Hua’er singing preserve cultural knowledge and ancestral traditions among ethnic minority communities in southern and southwestern China. However, their potential role as psychological resources remains insufficiently examined. This study investigated the association between cultural realization, psychological resilience, cultural identity, and meaning-making, considering the moderating roles of intergenerational transmission engagement and community cultural vitality.

**Methods:**

A convergent mixed-methods design was employed. A total of 520 participants from six communities representing four folk-song traditions completed standardized and newly developed instruments. Quantitative data were analyzed using confirmatory factor analysis and a moderated mediation model with a multilevel extension. Additionally, semi-structured interviews were conducted with 36 participants and analyzed using reflexive thematic analysis.

**Results:**

Cultural realization was positively associated with psychological resilience, both directly and indirectly through cultural identity and meaning-making. The indirect association through cultural identity was stronger among participants with higher engagement in intergenerational transmission. Community cultural vitality was positively associated with resilience; however, its cross-level moderating effect was not supported. Given the inclusion of only six communities, this finding remains inconclusive. Qualitative analysis identified four themes: ancestral dialogue, collective singing as a coping mechanism, living pedagogy, and fragile communal recovery.

**Discussion:**

The findings suggest that traditional folk songs may function as culturally embedded psychological resources by supporting cultural identity, meaning-making, and resilience. Nevertheless, the cross-sectional, self-reported design precludes causal conclusions, and the observed associations may partly reflect selection into singing. Further longitudinal and multilevel research involving more communities is needed to clarify these relationships.

## 1 Introduction

Psychological and emotional ecosystems are unique to indigenous and ethnic minority communities globally, in which mental health is part of the cultural, spiritual, and communal life (Ciofalo et al., 2022; O’keefe et al., 2022). Traditional folk song systems play an important role in this ecosystem overall, and specifically for the ethnic minority communities of the south and southwest of China, including the Zhuang, Dong, Miao and Hui/Hua’er communities (Xiaobin and Ku Wing, 2025; Wang et al., 2025). The art forms of Zhuang antiphonal singing, Dong Grand Song polyphony, Miao Feige, and Hua’er singing are not only artistic performances but also vehicles of the narration of ancestral history, moral guidance, and collective emotion, and they have long been seen by ethnomusicologists and cultural historians as living carriers of indigenous knowledge (Tang and Phaengsoi, 2024; Bao and Chuangprakhon, 2025; Wang et al., 2024). Several of these traditions have been designated as national or UNESCO-recognized intangible cultural heritage in recent years, and international interest in their cultural value is rising. However, folk song has not been generally examined in psychology as a psychological resource, and this is especially the case in the empirical study of indigenous well-being (Ning, 2023; Albacete-Maza et al., 2023). Throughout, ‘indigenous’ is used as an analytic term for long-established ethnic minority communities with distinctive ancestral song traditions; its meaning in the Chinese context is defined in Section 2.1.

Current scholarship on culture and mental health has focused on either broadly defined concepts of acculturation, ethnic identity, or cultural participation or has limited its scope to a few specific aspects of the relationship between culture and mental health, leaving the particular processes by which a musical-narrative tradition influences mental health largely unstudied and untested (Gustavson et al., 2021; Dingle et al., 2021; Xu et al., 2026). While there is a strong theoretical understanding that culture is foundational to indigenous mental health, there remains a significant gap between the theoretical understanding and evidence at the mechanism level to support culturally grounded interventions (Klassen et al., 2024; Sunderland et al., 2024; Scarfe et al., 2024). The present study is motivated by three limitations. First, much of the psychological literature on cultural heritage focuses on background demographic indicators rather than psychological pathways and routes through which cultural engagement has led to resilience, with the latter still poorly understood (Ozer et al., 2021; Eriksson and Contu, 2022; Narimani and Naeim, 2025; Naeim and Basharpoor, 2026). Second, intergenerational transmission has been described rather than analyzed, meaning that many of these song traditions are described as evidence of cultural continuity instead of as a context that potentially indicates when and for whom cultural engagement is protective; the lack of analysis is a critical omission as many of these song traditions come under active transmission pressure from rural-to-urban migration, language shift, and declining youth participation in seasonal festivals (Dockery, 2020; Hammood et al., 2025; Zhang et al., 2025; Tang and Phaengsoi, 2024; Li and Chaiyason, 2024; Yu and Choatchamrat, 2024). Third, while it is clear that any given individual’s experience of folk-song depends on a functioning collective infrastructure, the community-level conditions that enable folk-song practice are rarely codified as ecological moderators in addition to the psychological processes of the individual folk-singer (Branquinho et al., 2020; Azar et al., 2025; Xu et al., 2026). If these gaps are not bridged, the field has a difficult time creating culturally authentic and psychologically effective mental health and cultural-preservation efforts, especially in an era when many of these traditions are facing demographic and institutional challenges (Marques et al., 2021; Hammood et al., 2025; Xu et al., 2026).

What role does cultural realization of traditional Chinese folk song play in the psychological resilience of members of indigenous ethnic minority communities, and under what conditions? This is broken down into four questions. First, is cultural realization positively associated with psychological resilience, and is this association accounted for, at least in part, by indirect effects through cultural identity salience and meaning-making operating as parallel processes? Second, is the indirect effect through cultural identity stronger when engagement in intergenerational transmission is high, that is, when elder–youth transmission is actively under way? Third, does community-level cultural vitality moderate the relationship between individual cultural realization and resilience, beyond the individual-level mechanisms? Fourth, how do community members themselves describe the psychological role of folk song, and how do their accounts agree with, diverge from, or enrich the pathway model estimated in the statistical analysis?

Correspondingly, the study aims to develop and psychometrically validate an instrument measuring cultural realization and another instrument measuring intergenerational transmission engagement, which are relevant and responsive to folk-song-practicing indigenous communities; to test a parallel-mediation structural model in which cultural identity salience and cultural identity meaning-making transmit the effects of cultural realization on psychological resilience; to test first-stage moderated mediation and cross-level moderation in a multilevel structural framework, with community cultural vitality moderating the relationship between cultural realization and psychological resilience and intergenerational transmission engagement and the relationship between cultural identity salience and psychological resilience; and to triangulate these quantitative pathways with thematically analyzed interview data, integrated into the broader multilevel structural model, producing a culturally grounded and mechanistically specified account of indigenous psychological resilience relevant to the broader comparative aims of this Research Topic.

Scope and terminology. Three clarifications frame the study. First, the outcome examined quantitatively is psychological resilience (CD-RISC-10), with sense of coherence treated as meaning-making; well-being in the broader, relational sense used in Indigenous scholarship is examined through participants’ own accounts rather than with a dedicated well-being scale. Second, the design is cross-sectional and based on self-report, so relationships are described as associations, and indirect effects are statistical rather than causal (Section 5). Third, the key constructs (cultural realization, cultural identity, cultural belonging, cultural participation, and intergenerational transmission) are distinguished conceptually and operationally in Section 2.2 and Table 1.

**Table 1:** Conceptual definitions of key and related constructs and their operationalization in this study.

Construct	Conceptual definition	How it differs from cultural realization	Operationalization in this study
Cultural preservation / revitalization	Community-, institutional- or policy-level efforts to safeguard a tradition (heritage listing, archiving, festivals, teaching programs).	Collective and structural rather than a personal appraisal; a person can value a tradition that the community is not actively maintaining, and vice versa.	Closest analogue is community cultural vitality (level 2): festival frequency, heritage-language use and transmission institutions, from archival and observational indicators.
Cultural participation / engagement	Behavioral involvement in the tradition (singing, listening, attending festivals, learning).	Describes what a person does. Someone can participate without valuing the tradition, or value it without participating.	Participation type (performer, learner, listener-only) used for sampling and description (Table 3); frequency of participation was not measured separately.
Cultural identity (identity salience)	Exploration of, and commitment to, one’s cultural or ethnic group membership (Phinney and Ong, 2007).	Concerns who one is as a group member; realization concerns what this particular tradition means to the person. Identity is a proposed mediator, not an equivalent.	MEIM-R, 6 items (exploration and commitment).
Cultural belonging / connectedness	Affective attachment to, and sense of membership in, the community.	Directed at the community and its people; realization is directed at the tradition as a heritage object.	Not measured separately; addressed qualitatively (Themes 2 and 4).
Cultural realization	Subjective, evaluative and affective recognition of a folk-song tradition as personally meaningful, valued and continuous with ancestral practice.	Focal construct: appraisal of the tradition’s personal relevance, pride, and continuity with ancestors, beyond knowing about it or taking part in it.	Cultural Realization Scale (CRS), 12 items, 7-point scale.
Intergenerational knowledge transmission	Relational process through which songs, meanings and practices pass between elders and youth, with reciprocal teaching and learning.	An interactional process rather than a personal appraisal; modeled as a boundary condition (moderator).	Intergenerational Transmission Engagement Scale (ITE), 10 items: self-reported frequency, reciprocity and quality of elder–youth song teaching. Actual knowledge acquired was not assessed.
Meaning-making	Interpretive process through which adversity and life events become comprehensible, manageable and meaningful (Antonovsky, 1987).	A psychological process on which cultural realization may draw; not specific to music.	SOC-13 (sense of coherence), used as a proxy for meaning-making.
Psychological resilience	Capacity to adapt to and recover from adversity, conceptualized here as an individual-level outcome that is formed within culturally embedded, multigenerational contexts (Hammood et al., 2025; Kirmayer et al., 2011).	Outcome variable. Community-level resilience is not measured.	CD-RISC-10 (Chinese version).

## 2 Literature review and hypothesis development

### 2.1 Indigenous communities and indigenous well-being in the Chinese context

The term ‘indigenous’ needs explicit definition in the Chinese context. In international scholarship, indigenous peoples are usually identified by a cluster of features rather than a single criterion: historical continuity with a territory that precedes the modern state, a distinct language and body of ancestral knowledge, self-identification as a distinct people, and continuing collective institutions that carry that knowledge (Kirmayer et al., 2011; O’Keefe et al., 2022). The People’s Republic of China does not use ‘indigenous peoples’ as an official category; its framework recognizes 55 ethnic minority groups (shaoshu minzu) alongside the Han majority, and whether and how the international concept applies to them is debated. We therefore use ‘indigenous’ as an analytic rather than a legal or political label, for ethnic minority communities that (a) have a long-established presence in a homeland region, (b) maintain a distinct language or dialect and an oral or musical tradition that carries ancestral history, moral teaching and collective memory, (c) self-identify as members of the group, and (d) sustain community institutions of transmission such as song societies, festivals and elder–apprentice relationships. The Zhuang, Dong and Miao communities studied here fit these criteria most clearly. The Hua’er site in Linxia is a more heterogeneous case: Hua’er is sung across several groups in northwest China (including Hui, Tu and Salar communities), and Hui communities in particular are widely dispersed and Chinese-speaking. It is included because its community-held singing tradition satisfies criteria (b) to (d), but findings from this site are interpreted with this difference in mind, and ‘indigenous ethnic minority communities’ is used in the title to signal both the comparative framing and the Chinese administrative context.

The meanings and functions of the songs are shaped by local history. In these communities the songs were historically transmitted orally rather than through written notation, and were embedded in village life, ritual and festival, carrying genealogical, moral and emotional knowledge (Zhang et al., 2025; Yuan and Napier, 2024; Yu and Choatchamrat, 2024; Wang et al., 2024). They are now under pressure from out-migration, language shift and declining youth participation (Tang and Phaengsoi, 2024; Li and Chaiyason, 2024; Yu and Choatchamrat, 2024), while the question of who transmits the tradition, and how, is itself changing (Zhang et al., 2025). Well-being in this setting is therefore understood relationally, as embedded in ancestry, community and collective practice rather than only in individual states (Kirmayer et al., 2011; Ciofalo et al., 2022; O’Keefe et al., 2022). To keep community accounts distinct from researcher interpretation, the interview protocol was developed with community cultural custodians, participants’ own terms were retained as codes and in quotations, and findings were returned to participants in member-checking sessions (Section 3.6).

### 2.2 Conceptual definitions: cultural realization and related constructs

Cultural realization is defined here as a person’s subjective, evaluative and affective recognition of a folk-song tradition as personally meaningful, valued and continuous with ancestral practice; that is, the extent to which the tradition is ‘realized’ as a living part of one’s own world rather than merely known about or encountered. It is an appraisal-level construct. It concerns what the tradition means to the person, not how often the person takes part (cultural participation or engagement), who the person understands themselves to be as a group member (cultural identity), how attached the person feels to the community (cultural belonging or connectedness), what the community does to safeguard the tradition (cultural preservation or revitalization), or how knowledge moves between generations (intergenerational transmission). Table 1 sets out these distinctions and the way each construct was operationalized. Empirically, cultural realization and cultural identity were related but distinguishable: the CRS correlated 0.42 with MEIM-R cultural identity (observed scores) and 0.46 at the latent level, and the square root of each construct’s AVE exceeded its correlations with all other constructs (Table 2). Cultural belonging and connectedness were not measured separately, so their distinction from cultural realization is conceptual and, in part, qualitative (Themes 2 and 4) rather than psychometric; this is acknowledged as a limitation in Section 5.

**Table 2:** Fornell-Larcker criterion: inter-construct correlations and √AVE (Diagonal).

Construct	Cultural realization	Transmission engagement	Cultural identity	Meaning-making	Resilience
Cultural realization	0.77	0.42	0.46	0.36	0.42
Transmission engagement	0.42	0.79	0.35	0.1	0.27
Cultural identity	0.46	0.35	0.77	0.2	0.43
Meaning-making	0.36	0.1	0.2	0.77	0.37
Resilience	0.42	0.27	0.43	0.37	0.77

### 2.3 Theoretical framework and rationale for the proposed pathways

Psychological resilience is operationalized at the individual level, but it is theorized as culturally embedded and multigenerational rather than as a purely individual trait. Hammood et al. (2025) propose that, in indigenous communities, resilience is transmitted across generations through culture, spirituality, oral narratives and collective practices, and Kirmayer et al. (2011) similarly place relationships with community and ancestral knowledge at the center of indigenous resilience. The FS-CRM follows this view in three steps: it measures an individual-level outcome (resilience); it models the cultural and relational processes thought to give rise to it (cultural identity, meaning-making, and intergenerational transmission); and it situates these processes in a community-level context (cultural vitality). Resilience as a community-level phenomenon is not assessed and is not a claim of this article.

The two mediators draw on distinct bodies of theory. The identity pathway rests on ethnic identity theory, in which identity develops through exploration of and commitment to group membership (Phinney and Ong, 2007): when a person experiences a song tradition as valued and continuous with ancestors, it supplies content for that commitment, and a stable collective identity is in turn associated with psychological adjustment (Klassen et al., 2024; Ozer et al., 2021). The meaning-making pathway rests on salutogenic theory, in which a sense of coherence (comprehensibility, manageability and meaningfulness) supports coping (Antonovsky, 1987); the lyrics and narratives of folk songs carry moral teaching and collective memory that can serve as interpretive frames for adversity, in line with accounts of shared cognitive frameworks as foundations of well-being in collectivist societies (Narimani and Naeim, 2025). Cultural realization is placed upstream because it is the appraisal that makes a tradition available as a personal resource, while identity and meaning-making are proximal psychological processes through which that appraisal may be translated into coping capacity. Intergenerational transmission and community vitality are positioned as boundary conditions because multigenerational and ecological accounts hold that whether cultural resources reach psychological outcomes depends on the strength of the transmission relationship and on the community conditions that sustain practice (Branquinho et al., 2020; Hammood et al., 2025). This ordering is theoretically motivated, but alternative orderings are plausible, for example identity strengthening realization, and cross-sectional data cannot discriminate between them (Section 5).

Folk-song engagement may also differ from general social participation or community involvement, which is itself associated with well-being through belonging and social support. The FS-CRM proposes that folk-song practice adds mechanisms that are at least partly specific to its musical-narrative form: (a) narrative and textual content, as lyrics carry genealogies, moral guidance and history, supporting meaning-making; (b) dialogic, call-and-response structure, in which grief or worry can be sung and answered by others; (c) embodied and synchronous performance, in which coordinated breathing, movement and vocal blending may support belonging and emotion regulation (Dingle et al., 2021; Golden et al., 2021); (d) ritual and calendrical settings, such as festivals, that anchor communal return and renewal; and (e) language and vocal style as markers of group identity (Yuan and Napier, 2024). Some of these overlap with what any communal activity provides. Because the study did not compare folk-song engagement with other forms of community participation, it cannot show that the associations are specific to folk song; the mechanisms above are proposed, and the interview material in Section 4.2.6 is suggestive only.

The FS-CRM is specified as a directional model because theory suggests this ordering, but reverse and reciprocal paths are plausible. Individuals with a stronger cultural identity and greater resilience may be more likely to sing, learn and attend festivals, and may report greater cultural realization. Longitudinal evidence from Indigenous Australian families reduces concern about reverse causality for cultural transmission and children’s adjustment (Dockery, 2020), but the cross-sectional data here cannot separate selection from effect. Accordingly, associational language is used throughout, and the indirect effects are treated as tests of consistency with the proposed model rather than as demonstrations of causal mediation.

### 2.4 Empirical background and hypotheses

Within the last 5 years, research on the well-being of indigenous and ethnic minority groups has converged on an important finding: culture serves not only as a signifier of group identity but as a dynamic social and emotional tool that impacts the meaning of adversity, emotional response, and sense of self. There is also recent evidence of culturally informed practices across First Nations Australia, Native North America, Latin America and rural China that these practices are all linked with feeling strong in cultural identity, feeling better emotionally, and feeling more connected to community (Marques et al., 2021; Klassen et al., 2024; Xu et al., 2026). A parallel body of theory argues that resilience is better understood as culturally embedded and transmitted across generations than as a solely individual characteristic: Hammood et al. (2025) propose a theory of multigenerational resilience in which culture, spirituality, oral narratives and collective practices carry resilience from elders to youth in indigenous communities, and related integrative models in other collectivist and Islamic-Asian settings likewise place cultural meaning systems, social relationships and shared cognitive frameworks at the center of mental-health promotion (Naeim and Basharpoor, 2026; Narimani and Naeim, 2025). Because these frameworks were developed largely outside China, they are used here as conceptual resources rather than as evidence that the same processes operate in the communities studied. However, in this literature, traditional music (and folk song in particular) is relatively under-theorized compared to other cultural-heritage dimensions, including land-based practices or ceremony, and very little research has attempted to test a formal mediation-moderation model in which song-based cultural engagement is linked to resilience through identity and meaning-making processes. This recent evidence base is summarized to support the 10 hypotheses that underpin the Folk Song-Mediated Cultural Resilience Model (FS-CRM).

The constructs in the present study rest directly on the three song traditions that define the target communities, and on the view of cultural realization and intergenerational transmission as observable processes, as opposed to abstract variables, that are protested in varying ways. In a longitudinal case-study of Zhaoxing Dong Village, published in Annals of Tourism Research, Zhang et al. (2025) demonstrated that the process of knowledge transfer in the transmission of this tradition, which is characterized as an unaccompanied, culture-oriented polyphonic tradition, has evolved from internal knowledge transfer to external knowledge transfer, expanding who transmits the tradition, how, and the scope of transmission. In tandem with this, Tang and Phaengsoi (2024) visited school administrators, song teachers, cultural inheritors and students in Guangxi, and discovered that the incorporation of Grand Song teaching into the primary and secondary education curricula had a measurable impact on musical literacy and continuity, and that out-migration of young people and an ageing group of masters of song were identified as the main transmission risks for the tradition – providing empirical evidence that intergenerational transmission engagement is a dynamic rather than a static construct. Mao et al. (2023) also showed that the experience of Grand Song soundscapes raised listeners’ subjective heritage-value evaluations compared to listening to the same music without pictures, highlighting that affective and value-laden dimensions of folk songs, which are fundamental to the cultural realization construct, are empirically measurable and sensitive to mode of exposure. Li and Chaiyason (2024), in their field work study with folk art practitioners, cultural preservers and community leaders in the Guangxi Zhuang Autonomous Region (GXZAR), also concluded that the embedding of Liu Sanjie Ge Yao into formal and informal education was effective in improving folk-music literacy, and that the major threat to the continuity of the tradition was a generational gap in interest; Wu and Boonsrianun (2023) documented similar findings from their study of the Zoupo Festival, which involves a communal singing tradition, in the same region of Guangxi; this again confirmed the importance of a tradition of communal singing as a major transmission mechanism across neighbouring ethnic groups in this region. Meanwhile, for the Miao whose traditions of musical transmission are also said to be central to cultural identity and function as part of the ceremonies, Yu and Choatchamrat (2024) documented the gradual evolution of music education and transmission practices in Yunnan Province, highlighting how modernization and urbanization have increasingly threatened the ability of traditional teaching methods to remain effective. In an ethnomusicological study, Yuan and Napier (2024) conducted song-taxonomy analysis in Fenghuang County, which provided direct evidence of how the repertoire functions as an identity-signalling system at the community level, consistent with the cultural-identity-salience mediator proposed in the present model. These studies specific to China collectively support a qualitative record of the three core processes this study formalizes statistically as live, variable, and consequential, with the potential to be found in Zhuang, Dong, and Miao communities, thus lending a good deal of contextual justification for testing such processes simultaneously in one quantitative structural model.

The first strand of evidence is related to the relationship between cultural engagement and cultural identity. Modern identity studies have shifted from using ethnic identity as a demographic variable to viewing it as a process of negotiation and negotiation, which becomes solid as a result of active cultural engagement. In the present study, the capacity to integrate the indigenous and mainstream cultural identities, not simply the exposure to Ladakhi culture, was found to predict psychological adjustment in indigenous youth in the context of local and global university settings, as studied by Ozer et al. (2021), in the case of indigenous youth in Delhi. In tandem with this, Klassen et al. (2024) investigated self-continuity in the lives of rural Indigenous youth in Canada, focusing on how community and cultural contexts influenced the way youth experienced their coherence across time, which was strongly linked to their sense of identity stability. Empirically, Barrita and Wong-Padoongpatt (2023) showed that ethnic identity has acted as a boundary condition influencing the relationship between protective psychological resources and well-being outcomes in a study of 696 people of colour, thus directly providing methodological support for treating identity-related constructs as both a mediator and a moderator within a single structural model. Overall, the literature suggests that cultural realization, or the recognition of one’s folk-song heritage by oneself in a subjective and valued way, has been positively linked with cultural identity salience.

*H1*: The relationship between cultural realization and cultural identity salience is positive.

A second strand focuses on meaning making. In recent methodological studies, the salutogenic tradition originating from the sense of coherence construct of Antonovsky has been thoroughly revisited, and Eriksson and Contu (2022) reiterated the cross-cultural stability and multilingual validation of the meaningfulness dimension of the sense of coherence construct – that life challenges are worth facing – and its suitability to ethnic minority groups. Extending this logic culturally, Narimani and Naeim (2025) argued that the foundations of psychological well-being in non-Western, collectivist cultures are found in the interaction of cultural values, social relationships, and shared cognitive frameworks, and hence folk songs (which carry collective narratives and moral instruction) have provided just such a framework of meaning. In an empirical study of Yi, Miao, Dong, and Yao communities in southwestern China within the same Frontiers in Psychology Research Topic that underpins the present study, Xu et al. (2026) concluded that the involvement in traditional ecological and cultural practices was significantly related to emotional resilience (*r* = 0.78), and that oral transmission of cultural knowledge among elders and their offspring was repeatedly reported by participants as a source of coping and mental guidance. This aligns with the folk song as a means of making sense of things, in the Antonovskian sense.

*H2*: Meaning-making (sense of coherence) is positively correlated with cultural realisation.

A third group of work connects the constructs of identity and meaning-making with those of resilience. Gustavson et al. (2021) recently reviewed the general literature on music and mental health in a widely cited Translational Psychiatry scoping review, finding that music engagement was consistently linked with improvements in mental health outcomes such as QOL and decreases in internalizing symptoms, with a warning that the majority of current studies did not identify the psychological pathway through which this occurs. In a Frontiers in Psychology scoping review, Dingle et al. (2021) filled this gap by identifying identity, belonging and shared meaning to be the two most common explanatory pathways through which music in groups (such as singing) positively affects well-being, which is what this two-mediator structure predicts. In a large cross-national study conducted during the COVID-19 pandemic, Ferreri et al. (2021) also demonstrated that musical engagement was a form of emotion-regulation and coping, especially for those who showed high musical reward sensitivity, further supporting the direct effect of musical engagement on the concept of resilience. Sunderland et al. (2024) and the related review by Scarfe et al. (2024) reported on using Indigenist yarning methodology with Australian and Vanuatu First Nations musicians that intergenerational musical practice has functioned as a cultural determinant of health to strengthen indigenous identity and challenge deficit-related narratives of indigenous well-being. Similarly, Marques et al. (2021) suggested that the therapeutic cultural environment of traditional healing and expressive practices promotes well-being because they combine affirmation of identity with culturally relevant coping mechanisms. This convergence of general music-psychology findings with evidence in the indigenous domain supports both cultural identity salience and meaning-making as co-mediators between cultural realization–resilience, while a direct effect of cultural realization–resilience has been expected, as the benefits of engagement with folk-songs are plausibly provided by non-identity, non-meaning effects such as physiological arousal regulation and social bonding, documented in the broader music-engagement literature (Golden et al., 2021).

*H3*: The relationship between cultural identity salience and psychological resilience is positive.

*H4*: There is a positive relationship between Meaning-making and psychological resilience.

*H5*: Cultural realization is directly associated with psychological resilience after accounting for the mediators (partial mediation).

*H6a*: Cultural identity salience mediates the relationship between cultural realization and psychological resilience.

*H6b*: Meaning-making mediates the relationship between cultural realization and psychological resilience.

A fourth strand is the intergenerational transmission as a boundary condition and not just a result. Dockery (2020), in a quasi-experimental study using Australia’s Longitudinal Study of Indigenous Children, found some of the strongest evidence to date that the transmission of traditional culture (connection to country, kin and traditional knowledge) by parents was associated with later socio-emotional adjustment of children, which alleviated previously raised concerns about reverse causality in this literature. In a narrative review, Hammood et al. (2025) thus introduced the concept of “multigenerational resilience theory,” which states that resilience itself is passed down through the generations, in the ways that these cultural resources are culturally embedded in teaching, spiritual beliefs and practices, and collective memory, and that the strength of these transmission relationships between elders and young people influences the likelihood of these cultural resources being realized in individual psychological outcomes. Oral narratives and collective practices are central vehicles in this account, and folk songs are among the most concrete forms they take; the present study therefore treats folk-song singing as a specific instance of the culturally embedded transmission that multigenerational resilience theory describes in general terms. The direct empirical evidence comes from Xu et al. (2026), who found that the more often elders interacted with youth about ecological and cultural practices, the more positive the identity and resilience processes were among the youth and their communities, and that the experience of elders as informal counsellors was explicitly identified as influencing coping capacities. This body of work warrants consideration of intergenerational transmission engagement as a moderator to strengthen the pathway from cultural realization to identity, rather than just a predictor of such a pathway. Transmission is also not necessarily one-directional: younger singers may reinterpret, re-voice or extend repertoire, and who transmits a tradition, and how, is itself changing (Zhang et al., 2025). The transmission scale used here therefore includes reciprocal interaction, although the direct study of youth-led reinterpretation lies beyond its scope (Section 5).

*H7*: The relationship between cultural realization and cultural identity salience is moderated by intergenerational transmission engagement, with a stronger cultural realization–cultural identity salience relationship among higher transmission engagement individuals.

*H8*: Intergenerational transmission engagement (moderated mediation) is a conditional indirect effect of cultural realization to resilience through cultural identity salience.

Finally, a strand places these processes at the individual level in the context of community ecology. The authors of the cited studies, Branquinho et al. (2020), highlighted the importance of considering structures and collective participation opportunities at the community level, which are, in part, determinants of individual well-being outcomes, as articulated by Bronfenbrenner’s classic ecological systems theory. In an extension of this logic, Azar et al. (2025) reported on a distal risk factor: socio-cultural erosion at the community level, which encompassed the erosion of language, ritual and collective practice, and which was a risk factor for youth mental health, independent of individual-level cultural engagement. As the level of cultural vitality and the pathways of culturally realized adaptation to adversity vary greatly across different ethnic-minority communities in southern and southwestern China, it is theoretically justified to conceptualize culture as a level-2 moderator of the individual-level cultural-realization–resilience relationship in a multilevel framework, following recent demands from this literature to cross the levels of analysis and examine the relationship between culture and culturally realized adaptation to adversity in a multilevel framework (Xu et al., 2026; Hammood et al., 2025).

*H9*: Psychological resilience is positively related to community cultural vitality.

*H10*: Community cultural vitality is a moderating variable between individual cultural realization and psychological resilience, with an influence that is stronger in communities with greater cultural vitality.

Together, this literature clearly shows convergence and an identified gap. Conversely, convergence is the consistent identification across Australian First Nations, Native North American and Latin American ethnic-minority settings of both the importance of culturally-embedded practice for identity, meaning, and resilience, and the importance of intergenerational connections and community-level vitality for the strength of these benefits. The gap is identified in the absence of studies that (a) treat folk song as a cultural practice of interest, rather than discursive activity, (b) test parallel mediation by identity and meaning-making simultaneously instead of treating them as if interchangeable, and (c) model intergenerational transmission and community vitality as moderators at their relevant levels of analysis in a single integrated structural model. The FS-CRM, along with the 10 hypotheses outlined above, go directly to the root of the problem (see Figure 1).

**Figure 1:**
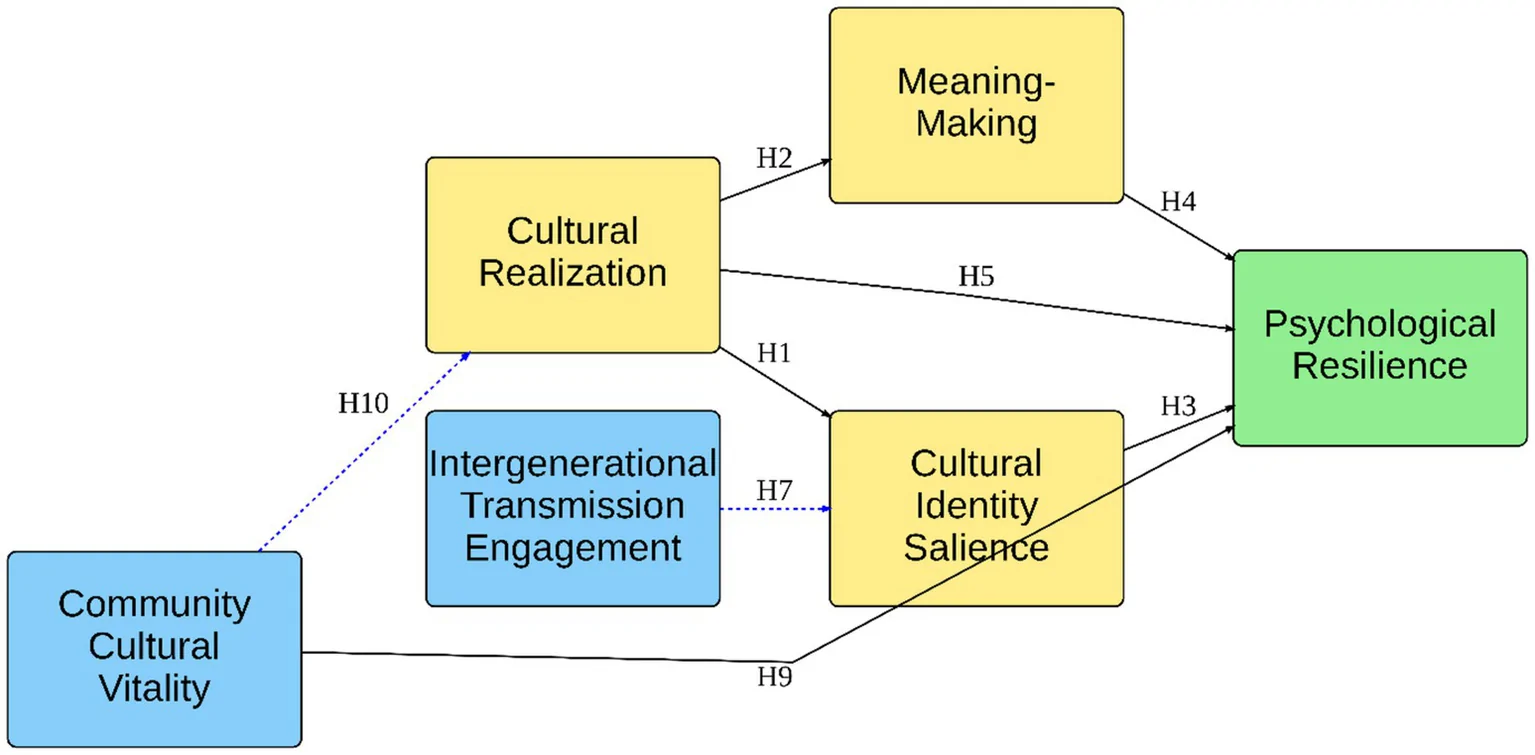
Conceptual framework diagram.

## 3 Research methodology

### 3.1 Research design and epistemological positioning

The research design used in this project is a convergent mixed-methods approach where both the cross-sectional quantitative survey and the qualitative strand that is embedded within ethnographic traditions have been employed. The quantitative approach has tested a moderated mediation structural model that assesses the relation between cultural realization and psychological resilience through cultural identity and meaning-making pathways. The design is pragmatist in orientation: both strands were given equal priority, were collected concurrently, and were analyzed separately before being integrated at the interpretation stage through a joint display (Fetters et al., 2013). The qualitative strand followed community-based and Indigenous research principles in which community members are treated as holders of knowledge rather than only as sources of data (Smith, 2012), and the quantitative findings are reported as associations because of the cross-sectional design.

### 3.2 Study context and sampling

The research was conducted within the ethnic minority communities of Southern and Southwestern China, which have folk song systems that are listed as their intangible cultural heritage: Zhuang antiphonal singing, Dong Kgal Laox polyphonic songs, Miao Feige traditions, and Hua’er singing in Hui, Tu, and Salar communities. The aforementioned communities were chosen due to their preservation of intergenerational traditions such as mentorship of elders, apprenticeships, and performance at festivals despite facing the challenges related to demography, including rural to urban migration and language shift. A stratified purposive sampling method was used during the research; stratification was based on participants’ generation (youth 18–29 years old, middle-aged people 30–54 years old, and elders over 54 years old), gender, and involvement in folk song culture (performers, learners, and only listeners). The snowball sampling approach through cultural bureaus and song-society (gefang) networks was then used to find the elders and other hard-to-reach respondents. From the *a priori* G*Power analysis conducted for the structural model that involves two mediators and one moderator (a small-to-medium effect size, f^2^ = 0.06, α = 0.05, and statistical power = 0.95), a quantitative sample of 480–560 participants was determined and increased by 20 percent to allow for non-responses and attrition through the various phases. After conducting data screening and administration of the survey, the final quantitative sample consisted of 520 participants, which was the achieved response rate, considering the non-useful questionnaires. For the qualitative research design, a purposive maximum variation sample of 36 participants was chosen to ensure equal representation from the different generational cohorts, ethnic communities, and participant types. The semi-structured interviews were conducted until theoretical saturation was reached. The six sites comprise four musical traditions (two Dong sites, two Zhuang sites, one Miao site and one Hua’er site; Table 3), so community and tradition are partly confounded. The power analysis addressed individual-level effects only; it did not take into account the number of communities (six), which constrains the cross-level test (Section 5).

**Table 3:** Sample characteristics (*N* = 520).

Variable	Category	*n*	%
Generational cohort	Mid-life (30–54)	196	37.7
	Elder (55+)	163	31.3
	Youth (18–29)	161	31.0
Gender	Male	262	50.4
	Female	258	49.6
Folk-song participation type	Learner	189	36.3
	Listener-only	187	36.0
	Performer	144	27.7
Community/site	Dong-Rongjiang	103	19.8
	Zhuang-Jingxi	94	18.1
	Hua’er-Linxia	89	17.1
	Miao-Taijiang	84	16.2
	Dong-Liping	78	15.0
	Zhuang-Napo	72	13.8

### 3.3 Conceptual model and its novelty

The present study proposes a unique integrative framework known as the Folk Song-Mediated Cultural Resilience Model (FS-CRM), which differentiates itself from the common acculturation-resilience model in that music and culture participation is not considered simply a symbolic form of ethnic identification but is seen rather as an actual psychological and sociological process that occurs along two parallel theoretical pathways: (a) Identity Consolidation through performing folk song, thus consolidating one’s ethnic identity, and (b) existential meaning-making through the use of lyrical content of songs for interpreting difficult situations (similar to Antonovsky’s sense-of-coherence concept). The unique aspect of this model is that it has three constructs not typically included together in previous research on well-being among indigenous people: (1) “cultural realization” – that is, how people subjectively experience and value their culture and heritage in a personal sense (as opposed to simply being knowledgeable or exposed to culture; defined and distinguished from related constructs in Section 2.2 and Table 1), (2) intergenerational transmission engagement (ITE) or how often and effectively elders teach youth – as a boundary condition (moderator) instead of a result, exploring whether the intensity of such transmission increases or makes the identity and meaning pathways effective, and (3) community cultural vitality (a measure on an ecological level of how often the community holds festivals, uses its language, and how institutions facilitate the maintenance of culture) as another boundary condition in a multilevel structural model, allowing the individual psychological pathways to be embedded in the community-level cultural environment. This mediation-moderation multilevel model, along with the development of a special psychometric scale for cultural realization, is the main methodological novelty of the study.

### 3.4 Measures and instrumentation

The measurement of cultural realization is based on the use of the Cultural Realization Scale (CRS), a new 12-item measure of cognitive, affective, and evaluative awareness of folk song tradition (for example, the perceived relevance to oneself, feelings of pride, and continuity with ancestors’ practice), which is rated on a 7-point Likert scale. The development of items for this scale is guided by the scale development manual (DeVellis, 2016) and the content validity is confirmed by expert rating (Lynn, 1986). Psychological resilience is measured with the 10-item Connor-Davidson Resilience Scale (CD-RISC-10; Connor and Davidson, 2003; Campbell-Sills and Stein, 2007) in its Chinese-validated version, which possesses good psychometric properties in Chinese samples (Yu and Zhang, 2007). Cultural identity salience, the first mediator, is measured with the adapted Multigroup Ethnic Identity Measure–Revised (MEIM-R; Phinney, 1992; Phinney and Ong, 2007), a two-factor (exploration and commitment) measure that demonstrates measurement invariance across ethnic groups (Brown et al., 2014). Meaning making, the second mediator, is quantified through the 13-item Sense of Coherence Scale (SOC-13), which stems from the salutogenic theory by Antonovsky (1987) and is psychometrically valid as determined by Antonovsky (1993), and whose cross-cultural relevance has been affirmed via a systematic review (Eriksson and Lindström, 2005). Engagement in intergenerational transmission is measured with a new scale, comprising 10 items that examine the frequency, mutual interaction, and quality of the teaching of songs by elders and youth, created using expert panel review and cognitive interviews with community participants, as outlined in procedures for scale development and piloting (DeVellis, 2016; Boateng et al., 2018). The ITE is a self-report index of perceived engagement in elder–youth song teaching (how often it occurs, how reciprocal it is, and how good the interaction is); it does not measure the amount of repertoire or knowledge actually transmitted, and is treated as an indicator of the strength of the transmission channel. Community cultural vitality, the level-2 moderator, is operationalized with a combination of archival and observational indicators (frequency of festivals, heritage language use in daily life, presence of transmission institutions in the community), aggregated at the village/site level, paralleling the ecological/community-level indicators used in other indigenous well-being studies (Kirmayer et al., 2011). The scales were forward- and back-translated into Mandarin and, where participants preferred, local minority languages (Brislin, 1970; International Test Commission, 2017), and cognitively pretested with 10–12 community participants per site.

Cultural and linguistic validity. Standardized instruments developed outside these communities carry assumptions that may not hold within them, and four steps were taken to address this. First, items were adapted through the translation and pretesting procedure above, following cross-cultural adaptation guidance (Beaton et al., 2000; International Test Commission, 2017), with wording checked for cultural appropriateness by community cultural custodians. Second, the MEIM-R, which was developed in North American multiethnic samples, was answered with reference to the participant’s own minority group, and the construct is labeled cultural identity salience in this article because the items address exploration of and commitment to one’s group. Third, the CD-RISC-10 has been validated in Han Chinese samples (Yu and Zhang, 2007) and the SOC-13 in many cultural settings (Eriksson and Lindström, 2005), but neither was previously validated in these communities, and resilience as an individual capacity may be narrower than local, more relational understandings of strength (Kirmayer et al., 2011). Fourth, within-sample evidence of measurement quality is reported in Section 4.1 (CFA fit, loadings, AVE, composite reliability and discriminant validity). Measurement invariance was tested across generational cohorts. Equivalence across communities, languages and traditions has not been established, and the findings should be read with this in mind (Section 5).

### 3.5 Statistical model and equations

The hypothesized relationships are estimated using structural equation modelling (SEM) with maximum likelihood estimation in R (lavaan) and cross-validated in Mplus. The parallel mediation component is specified as:

(cultural identity salience) (meaning-making / sense of coherence) (psychological resilience)

where denotes cultural realization and denotes resilience. The indirect effects are and, with total effect. Moderation by intergenerational transmission engagement (W) is introduced at the first stage (moderated mediation, Hayes’ Model 7 structure; Hayes, 2018):

with the conditional indirect effect expressed as, and the index of moderated mediation tested via bias-corrected bootstrap (5,000 resamples, 95% CI) to establish whether varies significantly across levels of. Community cultural vitality () is entered as a level-2 moderator in a multilevel SEM, with random intercepts for site and a cross-level interaction term predicting resilience, estimated via multilevel structural equation modelling (MSEM) to partition within- and between-community variance. Measurement invariance (configural, metric, scalar) is tested across generational cohorts before group comparisons, and common method bias is assessed using Harman’s single-factor test and a marker-variable approach (Podsakoff et al., 2003).

Between-community and tradition differences. Participants are nested in six sites representing four musical traditions. Site was modeled through random intercepts in the multilevel model; the site-level random-intercept variance (0.017) relative to residual variance (0.822) in that model (Table 4) implies a conditional intraclass correlation of about 0.02, that is, little residual variation in resilience lies between sites. The mediation and moderated mediation models were estimated at the individual level and did not model clustering explicitly, and tradition, ethnic group and site were not modeled as separate groups; whether the pathways differ across communities or traditions was therefore not tested (Section 5). Qualitative sampling was stratified to include all four traditions, and the illustrative quotations in Section 4.2 are drawn from each of them.

**Table 4:** Multilevel model predicting psychological resilience.

Parameter	Estimate	SE	*p*
Intercept	−0.001	0.066	0.9938
Cultural Realization (X)	0.376	0.04	0.0
Community Cultural Vitality (Z)	0.158	0.067	0.0188
X × Z (cross-level interaction)	0.035	0.041	0.3976
Random intercept variance (site)	0.017	—	—
Residual variance	0.822	—	—

### 3.6 Qualitative Strand and integration

The semi-structured interviews (60–90 min) explored participants’ subjective experience of learning, performing and transmitting folk songs, using an interview protocol developed with community cultural custodians to provide ecological validity. The interviews were conducted in the language participants preferred, audio recorded with permission, transcribed verbatim and analyzed using reflexive thematic analysis (Braun and Clarke, 2006, 2019) in NVivo. The initial deductive coding frame was based on the FS-CRM concepts, with additional inductive open coding for emerging themes (e.g., song as dialogue of ancestors, festival as ritual of resilience). To keep participants’ perspectives distinct from the researchers’ interpretation, three practices were followed. First, first-order codes retained participants’ own wording and were kept separate from second-order analytic codes and theme labels, so that labels such as ‘resilience practice’ are recognized as analytic constructs rather than participants’ terms. Second, quotations are presented before interpretation in Section 4.2, so that readers can judge the fit between the two. Third, all transcripts were independently double-coded by two coders, with disagreements resolved by discussion, and the themes were taken back to participants in member-checking sessions. The two strands were analyzed separately and then integrated at the interpretation stage by comparing path estimates with themes in a joint display matrix (Fetters et al., 2013), classifying each pathway as confirmed, explained, extended or contradicted by the qualitative findings (Table 5).

**Table 5:** Joint display matrix: integration of quantitative and qualitative findings.

FS-CRM pathway (quantitative)	Statistical result	Corresponding qualitative theme	Convergence assessment
Cultural Realization → Cultural Identity (a₁)	β = 0.416, *p* < 0.001	Theme 1: Song as Ancestral Dialogue	Convergent: confirms (a₁)
Cultural Realization → Meaning-Making (a₂); Meaning-Making → Resilience (b₂)	β = 0.336/0.233, *p* < 0.001	Theme 2: Collective Singing as a Resilience Practice	Convergent: confirms (a₂, b₂)
Transmission Engagement moderation (a₃); index of moderated mediation	β = 0.138, p < 0.001; index = 0.039, 95% CI [0.016, 0.065]	Theme 3: Elder-Youth Transmission as Living Pedagogy	Convergent: explains the moderation (a₃)
Community Cultural Vitality × Cultural Realization cross-level interaction (H10)	γ = 0.035, *p* = 0.398 (n.s.)	Theme 4: Festival and Ritual as Restorative, Yet Fragile, Communal Space	Divergent: extends the model (n.s. interaction; vitality as precondition)

### 3.7 Ethical considerations

This study subscribes to a community-based participatory research ethics philosophy suited to indigenous knowledge studies: it seeks ethical clearance from institutions, conducts oral and written consent procedures in the participant’s language, gives elder participants more time for consent and the opportunity for community witnesses, and stores and repatriates all song recordings to community culture agencies on agreed intellectual property terms. Reciprocity includes informing participating communities about findings in an easily understandable way and, if asked, giving recordings to community archives for heritage preservation purposes.

## 4 Results

### 4.1 Quantitative findings

The sample was well balanced, and participants were spread across the youth (31.0%), mid-life (37.7%), and elder (31.3%) age groups to provide good coverage of intergenerational perspectives. Gender distribution was almost even (50.4% male and 49.6% female), and the level of cultural engagement was reflected in the participation in folk-song traditions: learners (36.3%), listeners (36.0%) and performers (27.7%). Community representation was relatively even across the six research sites to capture variation at the individual and community levels in cultural experiences (Table 3).

The first goal was to create and test an indigenous folk-song community Cultural Realization Scale (CRS) and an Intergenerational Transmission Engagement Scale (ITE). Descriptive statistics revealed that the participants had relatively high cultural realization scores (M = 3.91, SD = 0.77) and cultural transmission engagement scores (M = 3.95, SD = 0.82). Similarly, the psychosocial functioning was generally positive, with positive scores for meaning-making (M = 3.98, SD = 0.76) and resilience (M = 3.07, SD = 0.76) and moderate scores for cultural identity salience (M = 2.94, SD = 0.78). The internal consistency coefficient (Cronbach’s alpha) of all measures ranged from 0.90 to 0.95, which is well above the recommended level of 0.70 and indicates high reliability for both newly created and adapted measures (Table 6).

**Table 6:** Descriptive statistics and internal consistency of study measures.

Construct	*N* items	M	SD	Min	Max	Cronbach’s α
Cultural Realization (CRS)	12	3.91	0.77	1.25	6.17	0.95
Intergenerational Transmission Engagement (ITE)	10	3.95	0.82	1.2	6.4	0.94
Cultural Identity Salience (MEIM-R)	6	2.94	0.78	1.0	5.0	0.9
Meaning-Making (SOC-13)	13	3.98	0.76	1.46	6.54	0.95
Psychological Resilience (CD-RISC-10)	10	3.07	0.76	1.1	4.9	0.94

The findings of the bivariate analyses confirmed the theoretical framework and showed significant positive correlations between the main constructs. There was moderate positive correlation for cultural realization with transmission engagement (*r* = 0.38), cultural identity (*r* = 0.42), meaning-making (*r* = 0.34), and resilience (*r* = 0.38). Likewise, cultural identity was positively related with resilience (*r* = 0.40) and meaning-making was correlated with resilience (*r* = 0.35). Community cultural vitality showed only weak correlations with the individual-level variables (*r* = 0.03 to 0.17, the strongest being with resilience), which is expected for a site-level indicator assigned to individuals and does not by itself indicate how vitality operates. Overall, the pattern of correlations observed was initial evidence of support for the proposed mediation model and was found to have adequate discriminant relationships between the study constructs (Table 7).

**Table 7:** Pearson correlations among study variables.

Variable	1.	2.	3.	4.	5.	6.
1. Cultural realization	1.0	0.38	0.42	0.34	0.38	0.04
2. Transmission engagement	0.38	1.0	0.31	0.1	0.23	0.03
3. Cultural identity	0.42	0.31	1.0	0.2	0.4	0.05
4. Meaning-making	0.34	0.1	0.2	1.0	0.35	0.05
5. Resilience	0.38	0.23	0.4	0.35	1.0	0.17
6. Community cultural vitality	0.04	0.03	0.05	0.05	0.17	1.0

The psychometric evaluation was very supportive of the validity of the newly created measurement model. All 51 observed indicators loaded significantly on their intended latent constructs, and factor loadings ranged from 0.721 to 0.817, which is above the recommended cut-off value of 0.70 for construct validity (Figure 2).

**Figure 2:**
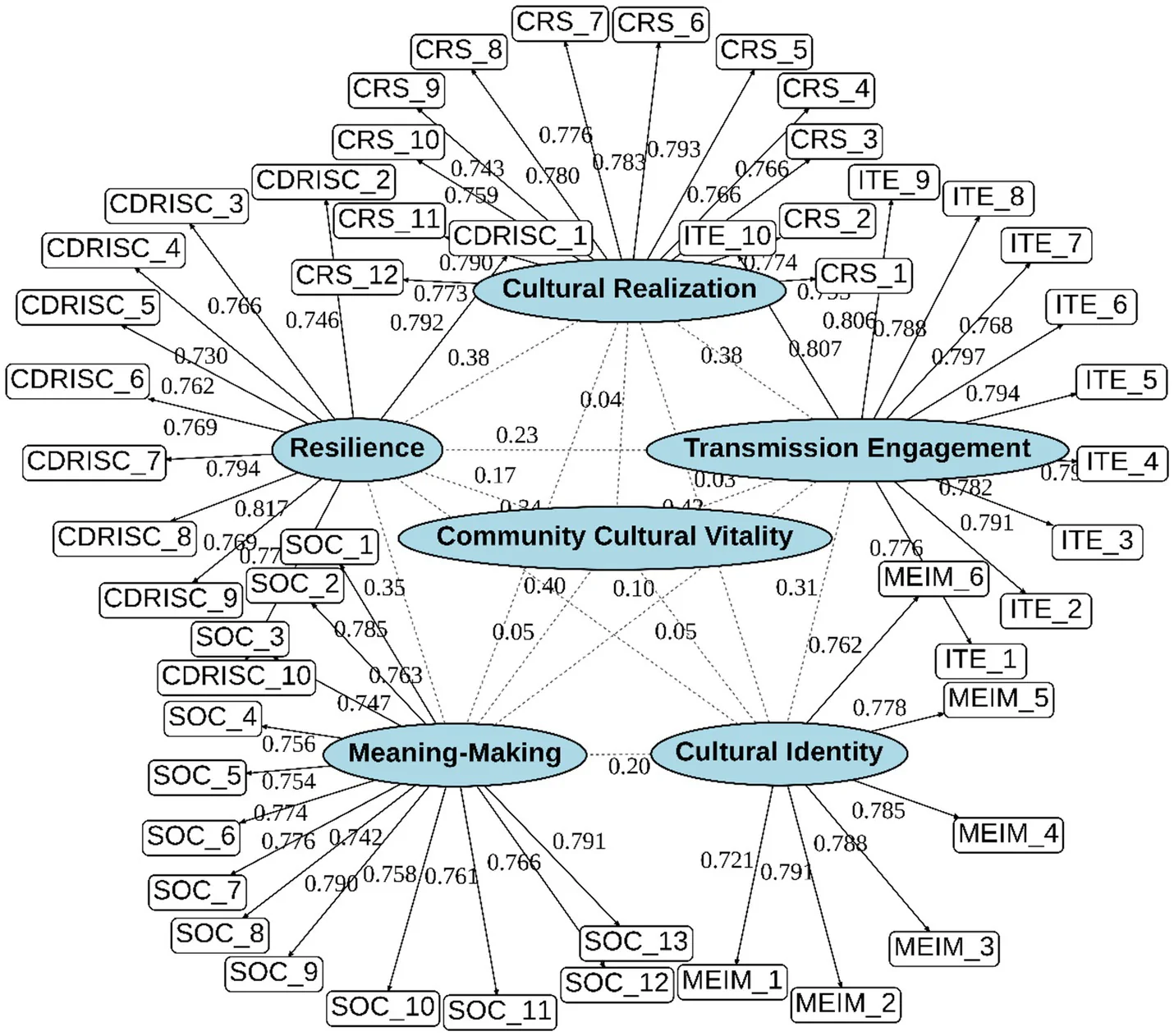
CFA measurement model.

The overall measurement model for five factors showed an excellent fit of the hypothesized measurement model with the observed data, with all the indices showing high values: χ^2^/df = 1.04, CFI = 0.997, TLI = 0.997, RMSEA = 0.009, GFI = 0.929, AGFI = 0.926, and NFI = 0.929 (Table 8). Also, the Average Variance Extracted values ranged from 0.588 to 0.625, and the Composite Reliability coefficients ranged from 0.898 to 0.949, thus indicating that each construct had more than 50% of variance explained in its indicators and high internal consistency (Table 9). The square roots of the AVEs were also higher than the inter-construct correlations for all constructs, meeting the Fornell-Larcker criterion and indicating good conceptual distinctiveness between the constructs (Table 2), thereby confirming the discriminant validity.

**Table 8:** Fit indices for the five-factor CFA measurement model.

Chi-square	df	Chi-sq/df	CFI	TLI	RMSEA	GFI	AGFI	NFI
1263.17	1,214	1.04	0.997	0.997	0.009	0.929	0.926	0.929

**Table 9:** Convergent validity: average variance extracted (AVE) and composite reliability (CR).

Construct	*N* items	AVE	Composite reliability (CR)
Cultural realization	12	0.6	0.947
Transmission engagement	10	0.625	0.943
Cultural identity	6	0.595	0.898
Meaning-making	13	0.588	0.949
Resilience	10	0.596	0.937

For the second research objective, cultural realization was significantly and positively associated with cultural identity salience (β = 0.416, *p* < 0.001) and meaning-making (β = 0.336, *p* < 0.001) as illustrated in the parallel mediation structural model. Meaning-making, in turn, was positively associated with psychological resilience (β = 0.233, *p* < 0.001) as did cultural identity (β = 0.282, *p* < 0.001). There was a significant direct effect of cultural realization on resilience (β = 0.186, *p* < 0.001), however, the total effect (β = 0.381) was significant, consistent with partial mediation. Bootstrap analyses confirmed that both mediators had statistically significant indirect effects, which were stronger with the pathway via cultural identity (β = 0.117, 95% CI [0.081, 0.157]) than with the pathway through meaning-making (β = 0.078, 95% CI [0.049, 0.113]). The results indicate that cultural realization of folk-song heritage is positively associated with resilience both directly and indirectly, through cultural identity and meaning-making. Given the cross-sectional design, these paths describe statistical associations consistent with the proposed model rather than established causal effects (Table 10).

**Table 10:** Standardized path coefficients for the parallel mediation model.

Path	B	SE	*p* / 95% CI
a1: Cultural Realization → Cultural Identity	0.416	0.04	0.0
a2: Cultural Realization → Meaning-Making	0.336	0.041	0.0
b1: Cultural Identity → Resilience	0.282	0.042	0.0
b2: Meaning-Making → Resilience	0.233	0.04	0.0
c’: Direct effect (Cultural Realization → Resilience)	0.186	0.043	0.0
c: Total effect (Cultural Realization → Resilience)	0.381	—	—
Indirect effect via Cultural Identity (a1*b1)	0.117	—	95% CI [0.081, 0.157]
Indirect effect via Meaning-Making (a2*b2)	0.078	—	95% CI [0.049, 0.113]

The third goal explored whether engagement in the intergenerational transmission process strengthened the cultural realization pathway. The first stage moderated mediation model results showed that the relationship between cultural realization and cultural identity was significantly moderated by transmission engagement (a₃ = 0.138, *p* < 0.001). That is, the effect of cultural realization through cultural identity onto resilience was progressively higher from low transmission engagement (β = 0.065, 95% CI [0.032, 0.102]) to average transmission engagement (β = 0.104, 95% CI [0.070, 0.141]) to high transmission engagement (β = 0.142, 95% CI [0.095, 0.195]). The significant index of moderated mediation (β = 0.039, 95% CI [0.016, 0.065]) indicated that the indirect association between cultural realization and resilience through cultural identity is stronger when elder–youth transmission engagement is higher (Tables 11, 12).

**Table 11:** Conditional indirect effects of cultural realization on resilience via cultural identity, at values of transmission engagement.

Level of transmission engagement (W)	Conditional indirect effect (a₁ + a₃W)·b₁	Bootstrap 95% CI
Low (−1SD)	0.065	[0.032, 0.102]
Mean (0)	0.104	[0.070, 0.141]
High (+1SD)	0.142	[0.095, 0.195]

**Table 12:** Summary of hypothesis tests.

Hypothesis	Path	Estimate	Result
H1	Cultural Realization → Cultural Identity Salience (a1)	β = 0.416, *p* < 0.001	Supported
H2	Cultural Realization → Meaning-Making (a2)	β = 0.336, *p* < 0.001	Supported
H3	Cultural Identity Salience → Resilience (b1)	β = 0.282, *p* < 0.001	Supported
H4	Meaning-Making → Resilience (b2)	β = 0.233, *p* < 0.001	Supported
H5	Cultural Realization → Resilience, direct (c’)	β = 0.186, *p* < 0.001	Supported (partial mediation)
H6a	Indirect effect via Cultural Identity (a1 × b1)	β = 0.117, 95% CI [0.081, 0.157]	Supported
H6b	Indirect effect via Meaning-Making (a2 × b2)	β = 0.078, 95% CI [0.049, 0.113]	Supported
H7	Transmission Engagement moderates Cultural Realization → Cultural Identity (a3)	β = 0.138, *p* < 0.001	Supported
H8	Index of moderated mediation (Transmission Engagement)	0.039, 95% CI [0.016, 0.065]	Supported
H9	Community Cultural Vitality → Resilience (level-2 main effect)	γ = 0.158, *p* = 0.019	Supported (tentative; six communities)
H10	Cultural Realization × Community Cultural Vitality (cross-level interaction)	γ = 0.035, *p* = 0.398	Not supported

Finally, it was determined whether community cultural vitality moderated the relationship between cultural realization and resilience in a multilevel analysis. Results also showed that psychological resilience was positively associated with community cultural vitality (γ = 0.158, *p* = 0.019) – meaning that people living in high cultural vitality communities reported higher levels of psychological resilience regardless of their own cultural realization levels. The hypothesized cross-level interaction between cultural realization and community cultural vitality, however, was not statistically significant (γ = 0.035, *p* = 0.398), suggesting that community vitality did not enhance or diminish the positive association between individual cultural realization and resilience (Table 4). Overall, nine of the 10 hypotheses were supported; H10, the cross-level interaction, was not (Table 12). Because only six communities were available, the level-2 estimates are imprecise, and H9 in particular should be treated as tentative (Section 5). Taken together, these findings are consistent with folk-song cultural realization being associated with resilience through complementary pathways (cultural identity and meaning-making), with the identity pathway stronger where intergenerational transmission engagement is higher, and with culturally vital communities showing somewhat higher average resilience (see Figure 3).

**Figure 3:**
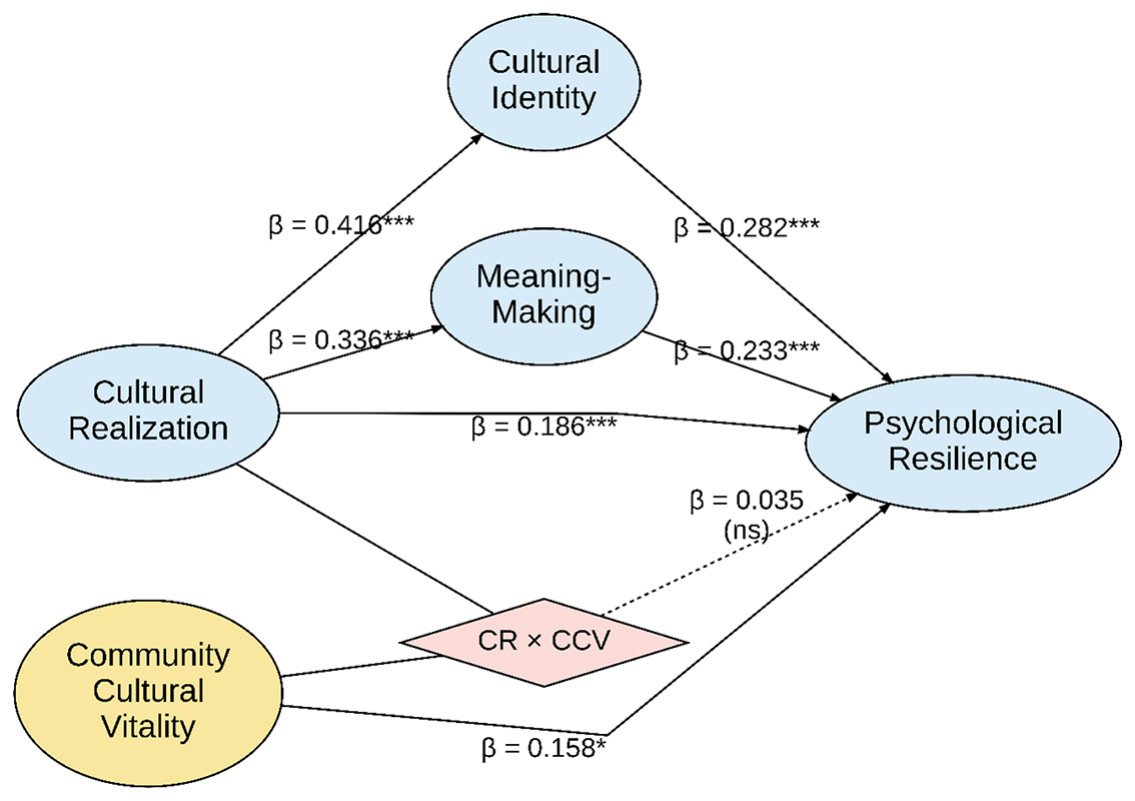
SEM pathway diagram.

### 4.2 Thematic analysis findings

A total of 36 semi-structured interviews were conducted (12 elders, 12 midlife, and 12 youth interviewees; distributed evenly among Zhuang, Dong, Miao, and Hua’er groups) and were coded using reflexive thematic analysis in NVivo. The transcripts were double-coded independently by two coders for 100% of transcripts according to deductive codes generated from the FS-CRM framework, supplemented with inductive open coding; the inter-coder agreement was calculated via Cohen’s kappa before reconciliation. Four themes emerged following consensus discussion (Table 13). Theme labels are the researchers’ analytic constructs; participants’ own words are retained in the quotations, and ‘resilience’ is an analytic label rather than a term most participants used (they spoke, for example, of ‘not carrying it alone’).

**Table 13:** Thematic coding summary: themes, initial codes, frequency, and inter-rater reliability.

Theme	Initial codes	Frequency (*n* references)	Cohen’s κ
1. Song as ancestral dialogue	Ancestral voice; lyrics as memory; felt continuity; pride in heritage; self-recognition in lineage	89	0.84
2. Collective singing as resilience practice	Grief expressed in song; communal coping; singing through hardship; emotional release; “not carrying it alone”	76	0.81
3. Elder-youth transmission as living pedagogy	Apprenticeship bond; oral correction; patience in teaching; reciprocal respect; duty to pass on	68	0.88
4. Festival and ritual as restorative (yet fragile) communal space	Festival as homecoming; ritual synchrony; village solidarity; anxiety over youth disengagement	61	0.79

#### 4.2.1 Theme 1: song as ancestral dialogue

The theme that emerged most often in the coding was of folk song as an experience of conversation and dialogue with ancestors. Over the generations, informants have spoken about lyrics as embodying the literal words and concerns of their ancestors, and thus singing as a way to temporarily live in the voice of their ancestors, rather than just in their memory. Elders particularly connected this feeling to a sense of “stability” in one’s own sense of self: if they knew the songs, they knew who they were, and several of them said they had a “calm confidence” in themselves when they were going through tough times in their lives. For the younger who were still learning the repertoire, they described the songs as a kind of theme that needed to be worked out, that they were still growing into, an aspirational version of the same theme, rather than a theme fully owned. “If I sing the old ones it’s not really me singing, but my grandmother’s grandmother.” — Dong elder, Participant 03, female, 71, from Rongjiang. The verses are not all known to me yet, but even those which I do know make me feel like I am not the first one to be here. — Participant 22, Zhuang youth, male, 21, Jingxi.

#### 4.2.2 Theme 2: collective singing as a resilience practice

The second theme involved singing as an active coping mechanism as opposed to a passive culture. Specific hardships, such as crop failure, bereavement, and the absence of family members from distant cities, were repeatedly discussed and described, using call-and-response singing at community meetings. Many informants commented that the nature of the songs themselves enabled a person to express grief or worry and then receive an answer from the group, something that they found to be more beneficial than expressing grief or worry alone. Participants in the middle years were mainly the ones who passed on the songs of suffering to younger singers and felt that this role was becoming endangered as fewer people met weekly for singing together, indicating that the resilience-building role of folk song is not only about an individual’s knowledge but also about the ongoing practice of the community to sing together. Participant 14, Hui/Hua’er, mid-life, female, 47, from Linxia: After my husband died, I could not have words, but when my line was answered by the women’s line, I felt the weight move. When the harvest was poor, we would sing instead of complain; it was not the person’s fault. — Participant 09, Miao elder, male, 63, Taijiang.

#### 4.2.3 Theme 3: elder-youth transmission as living pedagogy

A third theme centred around the particular interpersonal quality of intergenerational transmission, which was clearly separated from formal or classroom-based transmission. Embodied and relational teaching was repeatedly mentioned: How an elder was able to correct a breath or a tone during the moment of singing, instead of giving rules. Even in the absence of biological connection to the child, some youth and mid-life participants employed kinship language, referring to teachers as a “second grandmother” or a “song-father,” suggesting that the pedagogical relationship itself had an identity and emotion-laden dimension to it other than the transmission of the musical content. Elders, in turn, often defined teaching as a responsibility that comes with their own mortality, and with a responsibility to the future of the community—with several specifically connecting teaching to their own sense of life purpose that is continuing. She did not say that the words were incorrect; she only sang the line for me, softly, and I remembered my mouth. — Participant 27, Dong youth, female, 19, Liping. “If I don’t teach the young people, then the song has end with me. I have not let that happen.” — Participant 05, Zhuang elder, male, 74, Napo.

#### 4.2.4 Theme 4: festival and ritual as restorative, yet fragile, communal space

The last theme was that of seasonal festivals and ritual events in which the performance of folk songs was focused. The participants of these three generational age groups found that these meetings were restorative beyond the songs themselves, because these were opportunities for people who had migrated back to their families, and because of the aligned movement and breathing of group singing and because of the re-birthing of a sense of village solidarity that was not easily available in everyday life. This theme was accompanied throughout by an undercurrent of fear: many of the middle-aged and elderly participants openly expressed concerns about reduced attendance by young people at the festival, the diminishing number of voices capable of the performance of some of the verses over the years, and the fact that the festival’s restorative function depends upon a critical mass of participation that cannot be taken for granted. This tension made the festival at the same time a place of psychological renewal and a place of cultural concern for the future. “During the three days of the year, the whole village breathes together. I wait for this feeling all the year.” — Participant 18, Miao mid-life, female, 39 years old, Taijiang. “Fewer young people are coming to sing every spring; I am glad that we have the festival; I am worried for the next one.” — Participant 11, Hui/Hua’er elder, male, 68, Linxia.

#### 4.2.5 Integrating the themes and quantitative findings

Combined, the four themes form a logical sequence, progressing from individual meaning-making to collective sustainability, very similar to the quantitative FS-CRM pathways described above. Song as Ancestral Dialogue corresponds to the cultural identity pathway, and Collective Singing as a Resilience Practice to the meaning-making pathway, the two mediators of the statistical link between cultural realization and resilience. Elder-Youth Transmission as Living Pedagogy describes the interpersonal texture of the transmission engagement that strengthened the identity pathway in the structural model, and in doing so explains what ‘engagement’ meant in practice (relational, embodied and reciprocal). The fourth theme extended the model: participants described the psychological benefits of folk song as depending on a fragile, actively maintained collective infrastructure. This is compatible with the non-significant cross-level interaction, and suggests that community cultural vitality may be better understood as a precondition for the other three themes than as an independent moderator.

There is convergence in three of the four pathways (Table 5): the statistical mediators and moderator correspond clearly to the participants’ experiences of ancestral connection, communal catharsis, and teaching through embodiment. The exception is instructive rather than contradictory. In the case where there was no significant cross-level interaction in the multilevel model for community cultural vitality, the qualitative data explain why: the participants did not view cultural vitality as a variable that increases resilience linearly, but rather as an essential, fragile precondition. That is, on participants’ accounts, it is not enough to be more culturally vital; a minimum of collective practice has to be present for the other three processes to occur at all. This reading is *post hoc*. The null cross-level result may equally reflect limited power with six sites, little between-site variance in resilience, and a coarse site-level vitality index (Section 5), and the present data cannot separate these explanations from a threshold-type relationship.

#### 4.2.6 Musical, textual and contextual features discussed by participants

Reviewing the interview material for what participants said about the songs themselves, rather than only about their effects, shows that accounts of benefit were tied to particular features of the music and of the singing context (Table 14). Participants spoke of lyrics as carriers of ancestral words and memory (Theme 1); of the call-and-response form of the songs, in which grief or worry could be sung and answered by others (Theme 2); of the correction of breath and tone through embodied, in-the-moment teaching, and of learning a line by hearing it sung rather than by rule (Theme 3); and of the shared breathing and movement of group singing and the ritual setting of festivals (Theme 4). The quotations illustrating these features come from all four traditions (Table 14). By contrast, the codebook had no distinct codes for melody, rhythm, scale or mode, or language and dialect as such, so the analysis is sociocultural and performative rather than music-analytic, and it cannot show which melodic or rhythmic properties contribute to the reported benefits. Participants also tended to describe text, musical form, social experience and ritual setting as inseparable, for example when the weight of bereavement ‘moved’ as a sung line was answered by another group of singers (Participant 14). The data therefore do not allow the contributions of the music, the lyrics, the social experience of singing together and the surrounding cultural context to be apportioned; doing so would require designs that vary these elements separately.

**Table 14:** Musical, textual and contextual features identified in the interview data and their interpretive link to the model.

Feature	Evidence in interviews	Interpretive link to the model	Traditions in illustrating quotations
Lyrics and text as ancestral words	Theme 1; Participant 03 (Dong elder) and Participant 22 (Zhuang youth)	Meaning-making; identity continuity with lineage	Dong, Zhuang
Call-and-response form	Theme 2; Participant 14 (Hua’er, mid-life) and Participant 09 (Miao elder)	Shared emotional expression and relief; social support	Hui/Hua’er, Miao
Breath, tone and vocal technique learned through embodied demonstration	Theme 3; Participant 27 (Dong youth)	Identity- and emotion-laden teacher–learner bond; transmission engagement	Dong
Synchronous group singing (shared breathing and movement)	Theme 4; Participant 18 (Miao, mid-life)	Collective belonging; communal emotion regulation	Miao
Festival and ritual setting	Theme 4; Participant 18 (Miao) and Participant 11 (Hua’er elder)	Restorative communal space; return of migrants; fragility of participation	Miao, Hui/Hua’er
Melody, rhythm, scale or mode, language or dialect	No distinct codes in the codebook	Not analyzed	Not applicable

#### 4.2.7 Directions of transmission and challenges to transmission

The interview data speak to transmission in more than one direction, though unevenly. Elders described teaching as a duty tied to their own sense of purpose and mortality, and reciprocal respect was a recurrent code (Theme 3), suggesting that benefits and obligations ran in both directions. Younger participants described still-learning repertoire as something to grow into (Theme 1), and some used kinship terms for their teachers (Theme 3). However, the interview protocol did not ask younger participants specifically about reinterpreting, adapting or changing repertoire, so youth-led transformation of the tradition was not examined and remains an important gap (Section 5). The challenges to transmission that participants identified were reduced attendance by young people at festivals, fewer weekly gatherings for communal singing, the shrinking number of singers able to perform some verses, the absence of family members in distant cities, and strain on middle-aged singers who carry songs of hardship to the next generation (Themes 2 and 4). Participants linked the restorative function of festivals to a critical mass of participation that cannot be taken for granted.

## 5 Discussion, implications, and limitations

This study adds to the field of indigenous well-being by showing that cultural realization of Chinese folk-song heritage is associated with psychological resilience, directly and through overlapping cultural and psychological processes, which is consistent with folk songs functioning as an active resource rather than only as a cultural marker. In terms of the Folk Song-Mediated Cultural Resilience Model (FS-CRM), the current results are consistent with cultural realization being positively associated with resilience, both directly and through cultural identity salience and meaning-making, with engagement in intergenerational transmission moderating the identity pathway. This work is consistent with the increasing body of evidence suggesting that culture is more than just a symbol of ethnic belonging and a tool to interpret experiences, maintain continuity of self and form adaptation skills (Klassen et al., 2024; Xu et al., 2026; Marques et al., 2021). The current study integrates concepts of mediation, moderation and multilevel analysis in one framework and, thus, extends existing accounts of indigenous resilience, offering one of the first empirical tests of folk-song engagement within a multilevel, mixed-methods framework.

Two features of the design limit how far the results can be read as showing that folk-song engagement contributes to resilience, as opposed to being associated with it. First, the data are cross-sectional and self-reported, so the temporal order of cultural realization, identity, meaning-making and resilience is assumed from theory rather than observed. Second, the associations may partly reflect selection: people with a stronger cultural identity or greater resilience may be more likely to sing, learn and attend festivals, and to report that the songs matter to them, and the present analyses cannot separate this from an effect of singing itself. Some evidence fits the proposed direction, including participants’ retrospective accounts of singing as a way through bereavement and hardship (Theme 2) and longitudinal evidence from another Indigenous setting that transmission of cultural practice is associated with later adjustment (Dockery, 2020), but none of it rules out reverse or reciprocal paths, which are plausible if resilience is a reciprocal, intergenerational process (Hammood et al., 2025). The findings are therefore described as associations and the indirect effects as statistical, and we do not claim that folk-song participation develops resilience. Testing that claim would require longitudinal designs (for example, cross-lagged models across festival seasons), comparison with other forms of community participation, or intervention studies conducted in partnership with communities.

The development and validation of the scales for Cultural Realization and Intergenerational Transmission Engagement is a significant methodological innovation. The great reliability, high factor loadings, adequate convergent validity, discriminant validity, and very good fit of the measurement model suggest that both scales are reliable and valid measures of constructs that have been predominantly measured using qualitative methods or ethnography in previous studies. This means that the concepts of cultural realization and intergenerational transmission engagement are now operationalized in a form that can be used alongside qualitative work while retaining cultural content. Because the scales were developed and evaluated in the same sample used to test the structural model, replication in independent samples and tests of invariance across communities are needed before they are used comparatively. With the development of validated measures of these two concepts, there are now possibilities for comparative research among different indigenous groups and the growing demand for culturally relevant scales in resilience studies (Xu et al., 2026; Klassen et al., 2024).

The structural results are consistent with the theoretical idea that cultural identity and meaning-making act as complementary, rather than rivalling, routes for connecting cultural awareness to resilience. Cultural identity acted as the stronger mediator, implying that identifying oneself as a carrier of indigenous music heritage helps provide an enduring psychological grounding that allows individuals to be resilient in times of difficulty. Meaning-making also made important contributions, suggesting that folk music songs do not serve as mere markers of identity, but also serve as interpretive frames within which individuals interpret their suffering and retain hope. Qualitative themes supported these statistical relations, especially regarding the participants’ perceptions of ancestral conversations and collective singing as belonging, emotion regulation, and goal-oriented activities. Thus, these converging results are consistent with theoretical claims that music can support resilience not merely through providing psychological escape or distraction, but as a result of identity affirmation, sense of belonging, and cultural meaning systems (Dingle et al., 2021; Gustavson et al., 2021; Ferreri et al., 2021).

The music-specific dimension deserves separate comment. The statistical model captures folk-song engagement through a global appraisal (cultural realization), and the qualitative analysis was sociocultural, so the study cannot show which musical element (melody, rhythm, text, vocal style, language or ritual function) carries the reported benefits. What the interviews do indicate is that participants located benefit in particular features: lyrics as ancestral speech, which fits the meaning-making and identity pathways; call-and-response form, which turns private grief into something sung and answered; embodied vocal teaching, which makes the teacher–learner bond an identity-laden one; and the shared breathing and ritual setting of group singing, which fits accounts of music-based belonging and emotion regulation (Dingle et al., 2021; Golden et al., 2021; Table 14). These are hypotheses about mechanism rather than findings. They were not tested against general social participation, which would also be expected to foster belonging and support. Comparative designs that contrast singing with other communal activities, solo with group singing, and text-focused with melody-focused engagement would show how far the effects are specific to folk song.

The moderated mediation results indicate that the indirect association between cultural realization and resilience through cultural identity depends to some extent on the intensity of intergenerational transmission engagement. As transmission engagement was growing, the indirect effect of cultural realization on resilience through cultural identity grew increasingly powerful, underscoring the role of the interaction between elders and youth in maintaining both cultural continuity and psychological well-being. The qualitative data about teaching being relational, embodied, and responsibility-based gives important context to explain the reason for which the transmission plays the role of amplifying resilience instead of being a simple means of knowledge transfer within the culture. Such a perspective is consistent with recent theories according to which resilience is transmitted via cultural relations and practices (Dockery, 2020; Hammood et al., 2025; Xu et al., 2026).

The results can also be read within recent accounts that treat resilience as culturally embedded and intergenerational rather than solely individual. Hammood et al. (2025) argue that, in indigenous communities, resilience is transmitted across generations through culture, spirituality, oral narratives and collective practices. Four processes in that account have counterparts here. First, cultural meaning systems: the lyrics and narratives of folk songs supply interpretive frames for adversity, which corresponds to the meaning-making pathway and to Theme 1 (Narimani and Naeim, 2025). Second, social cohesion: collective, call-and-response singing and festivals provide shared emotional expression and village solidarity (Themes 2 and 4), in line with arguments that social relationships and shared cognitive frameworks underpin well-being in collectivist societies (Narimani and Naeim, 2025; Naeim and Basharpoor, 2026). Third, cultural transmission: the stronger identity pathway at higher transmission engagement, and the relational, embodied character of teaching (Theme 3), suggest that the quality of the transmission channel conditions whether cultural resources reach psychological outcomes. Fourth, intergenerational processes: elders described teaching as a source of purpose, suggesting that benefits may flow to transmitters as well as learners. The converse is the risk highlighted by work on socio-cultural erosion, in which the loss of language, ritual and collective practice weakens the supports on which culturally embedded resilience relies (Azar et al., 2025); participants’ worries about fewer singers and declining youth attendance (Theme 4) express the same concern. These frameworks come mainly from Iranian and Islamic-Asian settings and from other indigenous contexts, and they are used here to organize interpretation rather than as evidence of cross-cultural equivalence.

At the community level, cultural vitality was positively associated with resilience but did not moderate the relationship between individual cultural realization and resilience. This null result is best treated as inconclusive rather than as evidence of no moderation, for four reasons. First, the cross-level test rests on six communities; simulation work shows that group-level samples of this size give unstable estimates and biased second-level standard errors (Maas and Hox, 2005), and the power analysis in Section 3.2 addressed individual-level effects only. Second, little variance in resilience lay between sites (conditional ICC of about 0.02), leaving little for a site-level moderator to explain. Third, community cultural vitality was a coarse index, built from three archival and observational indicators and aggregated to six values, whose reliability and validity were not established; it also covaries with tradition and region, so its effects cannot be separated from other site differences. Fourth, the relationship may be non-linear, for example a threshold below which collective singing cannot be sustained, which a linear interaction term would not detect. The qualitative findings are compatible with this fourth possibility, since participants stressed the fragility of festivals and weekly singing and described vitality as a precondition for the other processes (Theme 4), but this reading is *post hoc*. The pattern is in line with ecological approaches that treat community resources as the wider context for individual resilience (Branquinho et al., 2020; Azar et al., 2025), and future work with a larger number of communities, a validated vitality measure and tests of non-linear effects is needed to evaluate it.

There are several possible practical implications for cultural preservation and policy, offered cautiously given the associational design. First, cultural preservation efforts might treat folk songs as potential health-promoting resources and not only as intangible heritage to be conserved, taking care that valuing songs for health benefits does not displace their ritual and cultural meanings or the community’s ownership of them. Second, education programs for minority communities could support folk-song teaching and involve elders as cultural educators. Third, community development programs could prioritize intergenerational activities, festivals and apprenticeships that sustain interaction between elders and youth. Fourth, folk-song practice might be explored as a component of culturally grounded mental-health programs; the present study did not test any intervention, and such programs should be co-designed with communities and evaluated prospectively.

Several limitations should be noted. First, the cross-sectional design does not permit causal inference or establish temporal order, and the associations may partly reflect selection, in that people with stronger identity and resilience may be more likely to engage with folk song; longitudinal studies are needed to establish the temporal sequence of cultural realization, identity formation, meaning-making and resilience. Second, all quantitative measures were self-reported, so common method variance and social desirability are possible, and Harman’s single-factor test and a marker-variable approach offer only partial protection (Podsakoff et al., 2003). Third, the Cultural Realization and Intergenerational Transmission Engagement scales were newly developed and evaluated in the same sample used to test the structural model, so their structure needs replication in independent samples. Fourth, the cultural and linguistic validity of the standardized instruments in these communities rests on translation, pretesting and within-sample evidence; measurement invariance across communities, traditions and language versions has not been established (Putnick and Bornstein, 2016), and the resilience instrument may not capture relational understandings of strength. Fifth, the study did not compare folk-song engagement with other forms of social or community participation, so it cannot show that the associations are specific to folk song, and related constructs such as cultural belonging, connectedness and frequency of participation were not measured separately from cultural realization.

Sixth, the community-level analyses are limited. Only six communities, representing four traditions, were sampled, so community and tradition are confounded; the mediation models did not model clustering explicitly, and the pathways were not compared across communities or traditions; and the level-2 estimates are imprecise. For example, the t ratio of 2.36 for H9 corresponds to p of about 0.08 if the degrees of freedom are set at the number of communities minus two, so H9 is best regarded as tentative and H10 as untested rather than disconfirmed. Seventh, the sample covers a limited set of Chinese folk-song traditions, the Hua’er site is a more heterogeneous case of an ‘indigenous’ community (Section 2.1), and caution is needed in generalizing to other indigenous groups. Eighth, the analysis did not separate the contributions of melody, rhythm, text, vocal style, social experience and ritual setting, and it did not examine younger generations’ reinterpretation or transformation of repertoire, so transmission was studied mainly as elder-to-youth teaching, with reciprocity captured only through the mutual-interaction items and the interviews. Finally, although the qualitative findings helped interpret the statistical model, future research using longitudinal mixed-methods designs, larger multilevel samples with more communities, validated community-level measures and cross-cultural comparison would clarify how indigenous musical traditions relate to resilience. Overall, despite these limitations, the study provides evidence consistent with the FS-CRM and suggests that traditional Chinese folk songs can be regarded as a cultural resource associated with indigenous psychological resilience.

## 6 Conclusion

This research provides evidence that cultural realization of traditional Chinese folk songs is positively associated with psychological resilience among members of indigenous ethnic minority communities, directly and through cultural identity and meaning-making, and that the identity pathway is stronger where intergenerational transmission engagement is higher. The Cultural Realization Scale and the Intergenerational Transmission Engagement Scale offer initial tools for measuring these constructs, although they require replication and cross-community validation. Community cultural vitality was positively associated with resilience but did not moderate the individual relationship in this study, a result that is inconclusive given only six communities. Combining structural modelling with thematic analysis suggests how folk song traditions may work as cultural memory resources, as identity formation processes, and as instruments of collective continuity. Because the design is cross-sectional and self-reported, the findings do not show that folk-song participation causes resilience. Apart from advancing the Folk Song-Mediated Cultural Resilience Model, this research highlights the value of recognizing indigenous musical culture as a cultural and psychological resource: the preservation and revitalization of folk song traditions matter not only for intangible cultural heritage but also, plausibly, for sustainable communities, strong intergenerational connections and culturally grounded approaches to well-being.

## Data Availability

The original contributions presented in the study are included in the article/supplementary material, further inquiries can be directed to the corresponding author.
